# Deletion of bZIP Transcription Factor *PratfA* Reveals Specialized Metabolites Potentially Regulating Stress Response in *Penicillium raistrickii*

**DOI:** 10.3390/jof11010072

**Published:** 2025-01-17

**Authors:** Anxin Zhang, Shu Zhang, Xinran Xu, Wen-Bing Yin

**Affiliations:** 1 State Key Laboratory of Microbial Diversity and Innovative Utilization, Institute of Microbiology, Chinese Academy of Sciences, Beijing 100101, China; zhanganxin19950802@163.com (A.Z.); fulisan12138@163.com (S.Z.); 2 Medical School, University of Chinese Academy of Sciences, Beijing 100049, China

**Keywords:** *Penicillium raistrickii*, *PratfA*, raistrilide A (**1**), tunicoidine F (**2**), oxidative stress

## Abstract

Fungal secondary metabolism (SM) is highly correlated with physiological processes that are typically regulated by pleiotropic regulators. In this study, we purposefully altered *PratfA*, a crucial regulator associated with oxidative stress in *Penicillium raistrickii* CGMCC 3.1066. After the knockout of *PratfA*, a novel polyketide (PK) raistrilide A (**1**) and the known nonribosomal peptide (NRP) tunicoidine (**2**) subsequently disappeared. Notably, compound **1** is a rare octaketone derivative and contains two unsubstituted *cis*-double bonds, demonstrating its unique biosynthetic mechanism. The knockout of *PratfA* resulted in the disappearance of **1**–**2** and greatly increased the susceptibility of Δ*PratfA* mutant strain to oxidative stress, rendering it nearly impossible to survive in such environments. At present, the *OE⸬PratfA* strain showed no phenotypic or oxidative stress sensitivity differences compared to the wild-type strain. Our findings highlight that the oxidative-stress-related transcription factor (TF) *PratfA* influences SM pathways in *P*. *raistrickii*. The manipulation of regulatory factors can guide the discovery of novel natural products (NPs).

## 1. Introduction

Fungi represent a significant source for the discovery of active natural products (NPs), including the well-known antibiotic penicillin, lipid-lowering drug lovastatin, and others [1,2]. External stimuli and environmental changes generate signals that trigger fungal cascades and multilevel regulatory networks to produce secondary metabolites (SMs) that act as “chemical weapons” [3,4]. Manipulating global regulatory factors in regulatory networks is an effective method of obtaining novel SMs [5,6,7]. For instance, the disruption of LaeB led to the discovery of eight novel silent SMs.

The bZIP transcription factors (TFs) are conserved evolutionarily across all eukaryotes and play crucial roles in organisms’ responses to environmental stressors [8,9]. Atf1 is an important bZIP TF that is essential for the formation of fungal resistant structural conidia and is also involved in oxidative stress [10,11,12]. *FvatfA* from the maize pathogen *Fusarium verticillioides* regulates vegetative and invasive growths and orchestrates oxidative and cell wall integrity stress defenses of *F*. *verticillioides*. The Δ*FvatfA* mutant was deficient in fumonisin production, resulting in decreased carotenoid and increased bikaverin yields [13]. Sterigmatocystin (ST), a mycotoxin, plays a dual role in fungal biology. It enables fungi to compete for space in nature and acts as a signaling molecule, regulating metabolic processes within fungi. Researchers have observed that the deletion of the bZIP TF *atfA* resulted in the termination of ST synthesis in *Aspergillus nidulans*, which is of significant interest for the study of fungal survival mechanisms and SMs in nature [14].

Here, we used a strategic approach involving manipulation of regulatory factors to discover novel natural products. We focused on a bZIP-type transcriptional factor named *PratfA*, which is involved in the response to oxidative stress in *Penicillium raistrickii*. Genetic manipulation of this factor was employed to further investigate *PratfA* responses to oxidative stress and discover a new natural product.

## 2. Materials and Methods

### 2.1. Strains, Media, and Culture Conditions

The fungal strain *P. raistrickii* CGMCC 3.1066 was purchased from China General Microbiological Culture Collection Center (CGMCC, Beijing, China). *P. raistrickii* CGMCC 3.1066 and its transformants were grown at 26 °C on Potato Dextrose Agar (PDA, Becton, Dickinson and Company, Franklin Lakes, NJ, USA) or Potato Dextrose Broth (PDB, Becton, Dickinson and Company, USA) with appropriate antibiotics as required. Rice medium was used for compound isolation and characterization.

### 2.2. Bioinformation Analysis

The local BLAST approach was employed to identify the *PratfA* homologous sequences within *P*. *raistrickii* CGMCC 3.1066. The entire genome sequence of *P*. *raistrickii* CGMCC 3.1066 was inspected to identify a gene encoding a protein homologous to the *Schizosaccharomyces pombe* Atf1 (Gene ID: 2540329). A single gene named *PratfA* (identity 57.1% and coverage 80.3%) was found to encode a protein the entire amino acid sequence of which was highly homologous to those of Atfl of *S. pombe*. The PrAtfA sequence has been uploaded to the GenBank database, and its accession number is PQ827448. *PratfA* DNA was PCR-amplified with the primers shown in Table S1.

### 2.3. The Construction of PratfA Deletion and Overexpression Cassette

The oligonucleotide sequences for PCR primers are listed in Table S1. The synthesis DNA synthesis of the primer was accomplished by Beijing Tsingke Biotech Co., Ltd. (Beijing, China). TransStart ^®^ FastPfu DNA polymerase (TransGen Biotech, Beijing, China) was used to perform PCR reactions from gDNA. Diagnostic PCR was performed using 2×T5 Super PCR Mix (Colony) (TSE005, Beijing Tsingke Biotech Co., Ltd., China).

The knockout cassette was designed according to the location of the target gene. The genomic DNA of *P*. *raistrickii* was used as a template to locate the target gene and then amplify its 5′ upstream and 3′ downstream length of about 1.2 kb. Meanwhile, pAG1-H3 was used as a template to amplify the *hph* DNA fragment with a length of about 2.2 kb. Furthermore, pYZM13 was used as a template to amplify the *P_gpdA_* primer fragment. All fragments were recovered, purified, and quantified. The fragments were assembled using the double-joint method, with three fragment molar ratios of 1:3:1 and a total DNA amount of 800 ng or less for PCR. The double-joint PCR product was used as a template to amplify the assembled full-length fragments using nested primers, and the fragments were recovered by final ethanol precipitation and solubilized in TE buffer and stored at −20 °C.

Gene overexpression assembly was performed as follows: The overexpression cassette was designed according to the location of the target gene. The genomic DNA of *P*. *raistrickii* was used as a template to locate the target gene and then amplified with the target gene upstream according to its expression direction as well as the length of the target gene of about 1.2 kb. At the same time, the *hph* DNA fragment was amplified in the same way as described above, and the plasmid pYZM13, which contained the strong promoter *P_gpdA_*, was used as a template for gene overexpression assembly. The gene overexpression component was amplified with approximately a 1.3 kb of the *P_gpdA_* DNA fragment. All fragments were recovered, purified, and quantified. The assembled and recovered fragments were stored at −20 °C.

### 2.4. Transformation of P. raistrickii

The spore solution of *P. raistrickii* was transferred to 30 mL of PDB medium, so that the final concentration of the spores was about 1 × 10^6^ spores/mL; this was incubated at 26 °C, at 200 rpm, for 12~14 h. When the length of mycelium was 3–5 times the diameter of spores as observed by the microscope, the incubation was stopped, and mycelium was collected. The germinated spore suspension was centrifuged at 4 °C, at 8000 rpm, for 10 min; the precipitated germinated spores were collected, resuspended in sterile water, washed twice (4 °C, 8000 rpm, 10 min), and centrifuged to discard the supernatant; then, the mycelium was collected and weighed to the wet weight.

The wall-lytic enzyme was treated with osmotic buffer. Then, it was filtered through a 0.22 μm filter to remove bacteria. After that, the enzymatic solution was prepared. The enzymatic conditions were set as follows: 10 mL of the enzyme solution contained 0.64 g vinotaste and 20 mg Yatalase. About 1.2 g wet weight mycelium was resuspended in 10 mL of enzyme solution. The enzyme solution was incubated at 28 °C and 100 rpm for 10 h. Gently transferring the protoplast suspension to a glass fiber tube, an equal amount of trapping buffer was added (0.6 M sorbitol, 0.1 M Tris-HCl, pH 7.0); then, this was centrifuged at 5000 rpm for 10 min at 4 °C with a horizontal rotor. The protoplasts obtained after centrifugation at 4 °C and 6000 rpm for 8 min were resuspended in an appropriate volume of STC buffer (1.2 M sorbitol, 10 mM CaCl_2_, 10 mM Tris-HCl, pH 7.5) for transformation.

Fragments were added to 100 μL protoplasts and incubated on ice for 50 min. Subsequently, 1.25 mL of 60% PEG solution (60% PEG4000, 50 mM CaCl_2_, 10 mM Tris-HCl, pH 7.5) was added to the protoplasts and incubated at room temperature for 20 min. The transformation system was mixed into melted 100 mL of PDSSA medium (3.9 g/L potato glucose agar (Becton, Dickinson and Company, USA), 20.5 g/L sucrose, 1 mL honey), mixed, and poured into 90 mm sterile flat dishes; this was placed in the incubator at 26 °C for 12–20 h. After fine hyphae were observed on the surface, PDA medium containing hygromycin B (60 μg/mL) resistance was added to completely cover the surface. All plates were incubated at 26 °C for 2–3 days. Single colonies were streaked out to PDA medium with hygromycin B. Transformants were grown in PDB medium. Genomic DNA extraction was carried out according to a previously described protocol [15]. The mutants were tested by diagnostic PCR using primers inside and outside of the corresponding gene, as listed in Table S1.

### 2.5. HPLC and LC-MS Analysis of SMs

For WT and *PratfA* manipulation strains, the same cultures used for stress measurement at 7 days were extracted for secondary metabolite assessment by HPLC analysis. Briefly, five 7 mm diameter agar plugs were taken from each plate and transferred to a 2 mL Eppendorf tube. The plugs were extracted with 1 mL ethyl acetate/methanol/acetic acid (89:10:1) each by sonication for 1 h at room temperature. The extracts were then dried completely at room temperature and dissolved in 500 μL methanol. Then, 20 μL extract was injected for HPLC analysis.

HPLC analysis was conducted with a Waters HPLC system (Waters e2695, Waters 2998, Photodiode Array Detector) using an XTerra MS C18 column (250 by 4.6 mm, 5 μm, Waters, Milford, MA, USA). Water with 0.1% (*v*/*v*) formic acid (A) and MeOH (B) was used as the solvent at a flow rate of 1 mL/min. For analysis of the crude extracts, substances were eluted with a linear gradient from 60 to 100% B in 20 min, washed with 100% (*v*/*v*) solvent B for 5 min, and equilibrated with 60% (*v*/*v*) solvent B for 5 min. UV absorptions at 210 nm were illustrated. Semipreparative purification on HPLC was performed on an SSI HPLC system (Teledyne SSI Lab Alliance Series III pump system and Series 1500 Photodiode Array Detector, Torrance, CA, USA) with an ODS column (C18, 10.0 by 250 mm, 5 μm, YMC, Kyoto, Japan) and a flow rate of 2.5 mL/min.

LC-MS analysis was performed on an Agilent HPLC 1200 series system equipped with a single-quadrupole mass-selective detector and an Agilent 1100LC MSD model G1946D mass spectrometer by using a Venusil XBP C18 column (3.0 by 50 mm, 3 μm, Bonna-Agela Technologies, Tianjin, China). Water (A) with 0.1% (*v*/*v*) formic acid and acetonitrile (B) was used as the solvent at a flow rate of 0.5 mL/min. The substances were eluted with a linear gradient from 5 to 100% B in 30 min, then washed with 100% (*v*/*v*) solvent B for 5 min, and equilibrated with 5% (*v*/*v*) solvent B for 10 min. The mass spectrometer was set in electrospray positive ion mode for ionization.

### 2.6. Large-Scale Fermentation, Extraction, Isolation, and Purification

A scale-up fermentation of the *P*. *raistrickii* with 3 kg of rice medium was performed, and the WT was cultured at 25 °C for 14 days. The mycelia and solid rice media were obtained and extracted with EtOAc (soaked in about 10 L EtOAc directly) three times. The extracted solutions were combined and evaporated under reduced pressure to yield the extracts (77.41 g). The extracts were subjected to silica gel column chromatography (CC) (200−300 mesh) and eluted with a dichloromethane−methanol gradient system (petroleum ether, 100:0, 100:1, 50:1, 20:1, 10:1, 5:1, 2:1, and 0:1, with each elution volume of 2 L) to yield fourteen fractions (Frs. 1–14). Fraction 7 was rich in **1**, **2**, and other compounds selected for further separation and purification by semipreparative high-performance liquid chromatography (*v*/*v*, CH_3_CN/H_2_O = 60/40), and the eluent was set at a flow rate of 2 mL/min.

### 2.7. General Experimental Procedures

UV spectra were recorded on a Shimadzu UV-2450 spectrophotometer (Shimadzu Corporation, Kyoto, Japan). NMR experiments were carried out on a Bruker AM-500 NMR spectrometer at 298 K. Structural assignments were made with additional information from g COSY, g HSQC, and g HMBC experiments. HRESIMS utilized an Agilent Accurate-Mass-QTOF LC/MS 6520 instrument (Agilent Technologies Inc., Santa Clara, CA, USA). Semipreparative HPLC was performed on an SSI HPLC system using an ODS column [HPLC (YMC-Pack ODS-A, 10 × 250 mm, 5 μm, 2 mL/min)] (Scientific Systems Inc., State College, PA, USA)

### 2.8. Stress Sensitivity Assays on Nutrient Agar Plates

To estimate the stress sensitivity of the mutant, 10^5^ freshly grown (7 days) conidia suspended in 5 μL 0.1% Tween 20 were spotted on PDA plates, which were also supplemented with one of the following stress-generating agents (concentrations and mechanisms of actions are given in parentheses): diamide (2 mM; triggers glutathione redox imbalance), menadione sodium bisulfite (MSB; 0.18 mM; increases intracellular superoxide concentrations), tert-butylhydroperoxide (tBOOH; 1 mM, accelerates lipid peroxidation), H_2_O_2_ (10 mM; increases intracellular peroxide concentrations), NaCl (1 M, salt ion), and sorbitol (2 M, osmotic stress). All stress plates were incubated at 26 °C up to 6 days. All treatments included three replicates. The experiment was repeated three times.

### 2.9. Statistical Analysis

For statistical analyses, data were analyzed using the GraphPad Instate software package, version 5.01 (GraphPad software Inc., San Diego, CA, USA) according to the Tukey–Kramer multiple comparison test at *p* ≤ 0.05. Mean values with asterisks are significant.

## 3. Results

### 3.1. Knockout and Overexpression of bZIP TF PratfA, and Their HPLC Analyses

**Figure 1:**
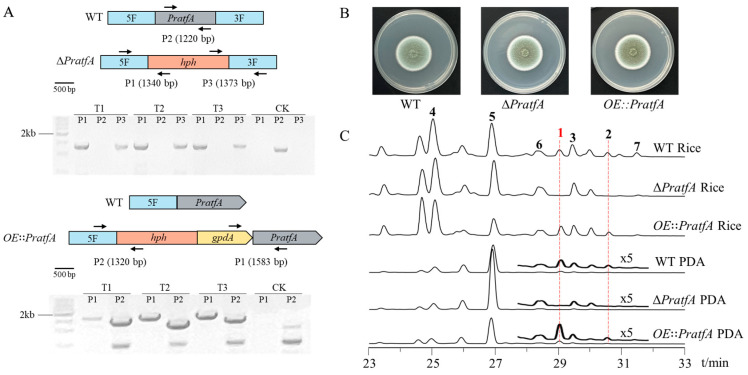
Knockout of bZIP TF *PratfA* leads to the disappearance of **1**–**2**. (**A**) PCR verification for *PratfA* gene deletion and overexpression in *P*. *raistrickii* wild-type strain (T1–T3 represent different Δ*PratfA*; CK represents *P*. *raistrickii*). The black arrows represent the primer positions used for validation. (**B**) The phenotypes of the *P*. *raistrickii* isogenic control strain, Δ*PratfA*, and the *OE⸬PratfA* mutants on a GMM plate at 26 °C for 6 days. (**C**) HPLC analysis of the crude extracts from the *P. raistrickii* isogenic control strain, Δ*PratfA*, and the *OE⸬PratfA* mutants in PDA and rice medium. The new compound is indicated in red.

**Figure 2:**
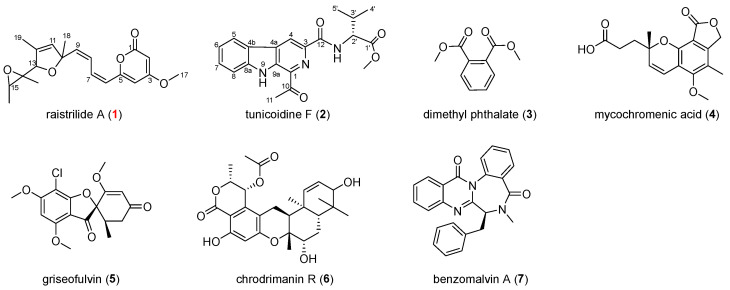
The structures of compounds **1**–**7**. The new compound is indicated in red.

In this study, we used a strategic approach involving manipulation of regulatory factors to discover novel NPs in *P. raistrickii* CGMCC 3.1066. The bZIP-type transcription factor AtfA of filamentous fungi is a putative direct homologue of Atf1, the “all-purpose” transcription factor of *S. pombe*, which regulates a broad spectrum of stress responses [16]. By searching the whole genome of *P. raistrickii* CGMCC 3.1066 using *S. pombe* AtfA amino acid sequence as a query, we identified a gene *PratfA* with the highest identity to *atfA* (identity 57.1% and coverage 80.3%). Subsequently, we aligned the PrAtfA sequence with the AtfA sequences of filamentous fungi to confirm the accuracy of the PrAtfA. These fungi were *P. chrysogenum* (identity 81.2% and coverage 97.0%), *A. nidulans* (identity 58.8% and coverage 90.0%), and *F. graminearum* (identity 47.7% and coverage 82.0%), respectively. Bioinformatic analysis indicated that the *PratfA* ORF, consisting of 1161 bp with no intron. Subsequently, we performed gene disruption and overexpression for *PratfA* in *P*. *raistrickii* based on homologous recombination as described (Figure 1A). The wild-type (WT), Δ*PratfA* (deletion of the gene sequence of *PratfA*), and *OE⸬PratfA* (insertion of the *P_gpdA_* promoter upstream of *PratfA*) mounts were cultured on a glucose minimal medium (GMM) at 26 °C for 6 days. Their phenotype did not change significantly (Figure 1B). Then, the WT, Δ*PratfA,* and *OE⸬PratfA* mounts were cultured in potato dextrose agar (PDA) and rice at 26 °C for 7 days. Clearly, the Δ*PratfA* mutant strain showed significant changes in the SMs profiles that **1** and **2** completely disappeared compared to *P*. *raistrickii* strain (Figure 1C, Figure 2 and Figure S1).

### 3.2. Identified Compounds from P. raistrickii CGMCC3.1066

The spore liquid of *P*. *raistrickii* were cultured in rice, and mycelia and solid rice medium were harvested, extracted, and subjected to repeated column chromatography to afford target compounds (Figure 2), of which raistrilide A (**1**) and tunicoidine (**2**) were differential compounds and dimethyl phthalate (DMP, **3**), mycochromenic acid (**4**), griseofulvin (**5**), chrodrimanin R (**6**), and benzomalvin (**7**) [17,18,19,20,21,22] were known compounds. Among them, raistrilide A (**1**) was identified as a novel toxin with two *cis*-double bonds.

### 3.3. Structural Characterization of Compound ***1***

Raistrilide A (**1**) was obtained as a yellow solid, giving the molecular formula C_20_H_24_O_5_ with 9 degrees of unsaturation on the basis of its ^13^C NMR and HRESIMS data (*m*/*z* 345.1693 [M + H]^+^; calcd 345.1702). The UV spectrum exhibited maximum absorption bands at *λ*_max_ 245 and 340 nm, revealing that **1** contained a conjugated double-bond system. The ^1^H, ^13^C, and HSQC NMR spectrum (Table 1) implied the following: four sp^3^ methyl groups [*δ*_H_ 1.66 (3H, s), *δ*_H_ 1.44 (3H, s), *δ*_H_ 1.35 (3H, d, *J* = 5.6 Hz), *δ*_H_ 1.22 (3H, s)], and their corresponding carbons individually at *δ*_C_ 12.9 (C-19), 26.6 (C-18), 13.7 (C-16), 11.4 (C-20); one sp^3^ methoxy group *δ*_H_ 3.79 (3H, s), and its corresponding carbon at *δ*_C_ 56.02 (C-17); nine submethyl groups, including seven sp^2^ submethyl groups, [*δ*_H_ 7.08 (1H, dd, *J* = 15.2, 11.0 Hz), *δ*_H_ 6.36 (1H, dd, *J* = 15.2, 11.0 Hz), *δ*_H_ 6.03 (1H, d, *J* = 11.0 Hz), *δ*_H_ 5.99 (1H, d, *J* = 11.0 Hz), *δ*_H_ 5.81 (1H, d, *J* = 2.2 Hz), *δ*_H_ 5.49 (1H, m), *δ*_H_ 5.44 (1H, d, *J* = 2.2 Hz)], 2 sp^3^ submethyl groups, [*δ*_H_ 4.20 (1H, s), *δ*_H_ 2.96 (1H, q, *J* = 5.6, 11.0 Hz)], and their corresponding carbons individually at *δ*_C_ 136.0 (C-7), 125.6 (C-8), 143.9 (C-9), 121.2 (C-6), 100.9 (C-4), 129.4 (C-11), 88.8 (C-2), 93.5 (C-13), 55.6 (C-15). In addition, the ^13^C NMR spectrum of **1** revealed two sp^3^ quaternary carbon: *δ*_C_ 89.0 (C-10), 60.9 (C-14); four sp^2^ hybridized carbons, including one carboxyl group *δ*_C_ 164.3 (C-1); two oxy-olefinic carbon *δ*_C_ 171.22 (C-3), 158.6 (C-5); one olefinic carbon *δ*_C_ 135.10 (C-13), which accounted for 6 degrees of unsaturation. The last three were assigned to three-ring system.

**Figure 3:**
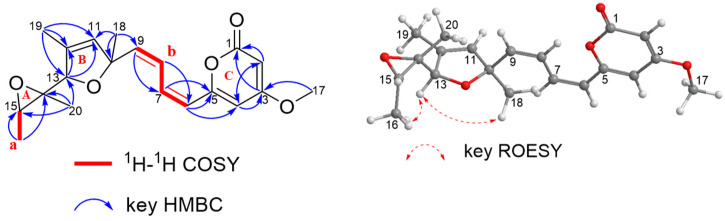
Key ^1^H−^1^H COSY, HMBC, and ROESY correlations for **1**.

Further analysis of the 2D NMR data was used to construct the complete structure of **1**. As shown in Figure 3, the ^1^H−^1^H COSY correlations revealed the presence of two structural segments a−b, as drawn with bold bonds (Figure 3). The COSY correlations (H-15/H-16) and HMBC correlations from H-16 to C-15, C-14, and from H-20 to C-13, C-14, and C-15 implied the presence of an ethylene oxide (ring A) based on the chemical shifts of C-14 and C-10 and from H-18 to C-10 and C-11 indicated the presence of a furan ring (ring B) and an olefinic bond (Δ^11(12)^). The key HMBC correlations from H-9 to C11 and from H-18 to C-9 and C-10 implied that C-9 and C-10 were directly connected. The COSY correlations (H-6/H-7/H-8/H-9) and the key HMBC correlations from H-19 to C-11, C-12, and C-13, from H-13 to C-11 and C-12, and from H-11 to C-12 and C-13 and correlations (H-6/H-7/H-8/H-9) and key HMBC correlations from H-9 to C-8 and from H-8 to C-6 and C-7 hinted at the presence of segment b. C-4, C-5, and C-6 were directly connected, which was supported by the key HMBC correlations from H-6 to C-5 and C-4 implied. The key HMBC correlations from H-4 to C-2, C-3, and C-5, from H-2 to C-1, C-3, and C-4 together with the chemical shifts of C-1 and C-5 concluded that there was a *α*-pyrone ring (ring C) in **1**. The chemical shift of C-3 and the key HMBC correlation from H-17 to C-3 implied the methoxy group was located in the position of C-3. Therefore, the planar structure of **1** was established as shown in Figure 3. In addition, the geometries of the double bonds (Δ^6^ and Δ^8^) were established as *cis*-configuration based on the coupling constant [Δ^6^, *J* = 11.0 Hz, and Δ^8^, *J* = 15.1, 11.0 Hz, *δ*_H_ 6.03 (1H, d, *J* = 11.0 Hz), and *δ*_H_ 7.08 (1H, dd, *J* = 15.2, 11.0 Hz)]. In the ROESY spectra of **1**, the correlations of H-16/H-13/H-18 (Figure 3), suggested they were cofacial. Structurally, Compound **1** is a rare octaketone derivative and contains two unsubstituted *cis*-double bonds, demonstrating its unique biosynthetic mechanism.

### 3.4. Oxidative Stress Sensitivity of P. raistrickii WT, ΔPratfA, and the OE⸬PratfA Mutants

**Figure 4:**
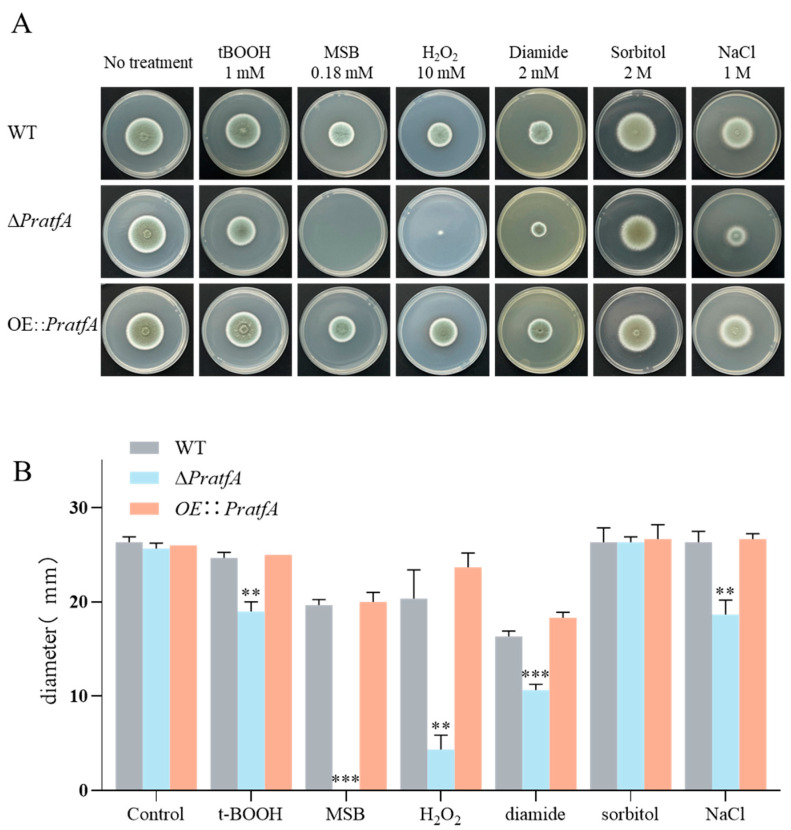
Oxidative stress sensitivity of the *P*. *raistrickii* isogenic control strain, Δ*PratfA*, and the *OE⸬PratfA* mutants. (**A**) The oxidative stress tolerances of the mutants were tested on a GMM plate. A quantity of 10^5^ freshly grown conidia was spotted on GMM, which was supplemented with one of the stress-generating agents: 1.0 mM *t*BOOH, 0.18 mM MSB, 10 mM H_2_O_2_, 2 mM diamide, 2 M sorbitol, and 1 M NaCl. The stress plates were incubated at 26 °C for 6 days. (**B**) The mean colony diameter values of *P. raistrickii* isogenic control strain, Δ*PratfA*, and the *OE⸬PratfA* mutants with different stress agents. ** *p* value < 0.01, *** *p* value < 0.001.

Next, we would like to continue exploring the impact of *PratfA* on the oxidative stress response. A quantity of 10^5^ freshly grown conidia was spotted on GMM, which was supplemented with four stress-generating agents: 1.0 mM *tert*-butylhydro-peroxide (*t*BOOH), 0.18 mM menadione sodium bisulphite (MSB), 10 mM H_2_O_2_, 2 mM diamide; two osmotic stress conditions: 2 M sorbitol and 1 M NaCl. The stress plates were incubated at 26 °C for 6 days, the colony diameters were measured and the percentage growth inhibition was calculated (Figure 4A,B). The growth status of the three strains remained consistent with colony diameters of 26 ± 0.5 mm under the GMM culture conditions without any stress inducer. However, the growth of all three strains was negatively affected under the four oxidative stress conditions.

There was no significant difference in growth rate between WT and *OE⸬PratfA* under four stress-generating agents and 1 M NaCl. The Δ*PratfA* strain exhibited less growth change compared to WT, and demonstrated a little sensitivity to *t*BOOH, sorbitol, and NaCl stress. In contrast, the Δ*PratfA* strain exhibited huge sensitivity to MSB, H_2_O_2_, and diamide stress, resulting in its inability to grow in such environment. The mean colony diameter values of WT and Δ*PratfA* strain were 19.9 mm and 0 mm under MSB stress, respectively. The mean colony diameter values of WT and Δ*PratfA* strain were 20.3 mm and 4.3 mm under H_2_O_2_ stress, respectively. The mean colony diameter values of WT and Δ*PratfA* strain were 16.3 mm and 10.7 mm under diamide stress, respectively (Figure 4A,B). In summary, knockout of Δ*PratfA* resulted in the disappearance of **1**–**2**, which in turn drastically increased the susceptibility of Δ*PratfA* mutant strain to MSB, H_2_O_2_, and diamide stress, rendering it nearly impossible to survive in these environments.

## 4. Discussion

Compounds produced under the control of global regulatory factors often exhibit excellent biological activity, especially those regulated by the bZIP TFs which are associated with oxidative stress. Therefore, compounds regulated by bZIP TFs are often closely related to fungal oxidative stress. In particular, we focused on one of the bZIP TFs, namely *PratfA*.

Subsequently, we identified seven compounds through knockout and overexpression of bZIP TF *PratfA* in *P*. *raistrickii*, including two potential toxins (**1**–**2**) that may be regulated by fungal oxidative stress. Compound **1** is a rare octaketone derivative and contains two unsubstituted *cis*-double bonds, demonstrating its unique biosynthetic mechanism, while compound **2** is a NRP compound. Compared with the WT, the Δ*PratfA* mutant strain exhibits higher oxidative and salt ion stress sensitivity. The two potential toxins **1** and **2**, which we discovered, are closely related to the environmental stress response ability of *P*. *raistrickii*. These findings contribute to further research on the function and regulatory mechanisms of bZIP TF *PratfA*, providing a crucial reference for the universal application of the strategy of manipulating TF to discover novel NPs.

## Figures and Tables

**Table 1:** ^1^H (500 MHz) and ^13^C NMR (125 MHz) data of **1** (*δ* in ppm).

Position	*δ*_C_ (Type)	Type	*δ*_H_ (Type, *J* in Hz)
1	164.3, C1	C	/
2	88.8, C2	CH	5.44, d (2.2)
3	171.2, C3	C	/
4	100.9, C4	CH	5.81, d (2.2)
5	158.6, C5	C	/
6	121.2, C6	CH	5.99, d (11.0)
7	136.0, C7	CH	7.08, dd (15.2, 11.0)
8	125.6, C8	CH	6.36, dd (15.2, 11.0)
9	143.9, C9	CH	6.03, d (11.0)
10	89.0, C10	C	/
11	129.4, C11	CH	5.49, m
12	135.1, C12	C	/
13	93.5, C13	CH	4.20, s
14	60.9, C14	C	/
15	55.6, C15	CH	2.96, q (5.6)
16	13.7, C16	CH_3_	1.35, d (5.6)
17	56.0, C17	CH_3_	3.80, s
18	26.6, C18	CH_3_	1.44, s
19	12.9, C19	CH_3_	1.66, s
20	11.4, C20	CH_3_	1.22, s

NMR data for **1** were recorded in CDCl_3_.

## Data Availability

The original contributions presented in the study are included in the article/Supplementary Materials, further inquiries can be directed to the corresponding authors.

## Supplementary Materials

The following supporting information can be downloaded at: https://www.mdpi.com/article/10.3390/jof11010072/s1, Table S1: The primers used in this study; Table S2: Recombinant strains plasmids used in this study; Figure S1: The HPLC analysis of WT, Δ*PratfA*, and the *OE⸬PratfA* mutants; Figure S2: ^1^H-NMR spectrum (500 MHz) of raistrilideA (**1**); Figure S3: ^13^C-NMR spectrum (125 MHz) of raistrilideA (**1**); Figure S4: HSQC spectrum of raistrilideA (**1**); Figure S5: COSY spectrum of raistrilideA (**1**); Figure S6: HMBC spectrum of raistrilideA (**1**); Figure S7: ROESY spectrum of raistrilideA (**1**); Figure S8: UV spectrum of raistrilideA (**1**); Figure S9: ESI mass spectrum of spectrum of raistrilideA (**1**); Figure S10: High resolution mass spectrum of spectrum of raistrilideA (**1**).
