# Supplementary material for: Deletion of bZIP Transcription Factor PratfA Reveals Specialized Metabolites Potentially Regulating Stress Response in Penicillium raistrickii
Source: J Fungi (Basel). 2025 Jan 17;11(1):72. doi: 10.3390/jof11010072 (PMC11766536; doi:10.3390/jof11010072)
Supplement: Supplementary file 1 [file jof-11-00072-s001.zip › jof-3396150-supplementary.pdf]

Supplementary Material

Deletion of bZIP transcription factor *PratfA* reveals specialized metabolites potentially regulating stress response in *Penicillium raistrickii*

Anxin Zhang [1,2†], Shu Zhang [1†], Xinran Xu [1,2,*], and Wen-Bing Yin [1,2,*]

[1] State Key Laboratory of Microbial Diversity and Innovative Utilization, Institute of Microbiology, Chinese Academy of Sciences, Beijing 100101, PR China; zhanganxin19950802@163.com (A.Z.); fulisan12138@163.com (S.Z.)

[2] Medical School, University of Chinese Academy of Sciences, Beijing 100049, PR China.

* Correspondence: yinwb@im.ac.cn, ORCID: 0000-0002-9184-3198; xuxr@im.ac.cn, ORCID: 0000-0003-3060-8554

† These authors contributed equally to this work

This PDF file includes:

Tables S1 to S2

Figures S1 to S10

Table of contents

1. **Supplementary Tables** 4

Table S1 The primers used in this study 4

Table S2 Recombinant strains plasmids used in this study 5

2. **Supplementary Figures** 6

Figure S1 The HPLC analysis of WT, Δ*PratfA*, and the *OE*::*PratfA* mutants 6

Figure S2 ^{1}H-NMR spectrum (500 MHz) of raistrilideA (**1**) 7

Figure S3 ^{13}C-NMR spectrum (125 MHz) of raistrilideA (**1**) 8

Figure S4 HSQC spectrum of raistrilideA (**1**) 9

Figure S5 COSY spectrum of raistrilideA (**1**) 10

Figure S6 HMBC spectrum of raistrilideA (**1**) 11

Figure S7 ROESY spectrum of raistrilideA (**1**) 12

Figure S8 UV spectrum of raistrilideA (**1**) 13

Figure S9 ESI mass spectrum of spectrum of raistrilideA (**1**) 14

Figure S10 High resolution mass spectrum of spectrum of raistrilideA (**1**) 15

Supplementary Tables

Table S1 The primers used in this study

Primer	Primer sequence（5'-3'）	Description
atf-5F-F	GAGTGATATGATTGGGCTGACAC	Up flanks' amplification for *PratfA* deletion
atf-5F-R	GCTCCTTCAATATCATCTTCTGTCGAGGCCTGATCATCGATGCGAGGAACCCGGCTCTG	Up flanks' amplification for *PratfA* deletion
atf-3F-F	ACTCTCCTATGAGTCGTTTACCCAGAATGCACCGCTGTGCTTTTTTGATGTATAATTGC	Down flanks' amplification for *PratfA* deletion
atf-3F-R	CTGTGATTGTCGTTGGAGTTGAC	Down flanks' amplification for *PratfA* deletion
atf-nest-F	CTTCCTATAGAAACCTCTGCTCTACG	knockout *PratfA* cassette
atf-nest-R	GCATACATACAGGACGCGTAC	knockout *PratfA* cassette
atf-RT-F	GTAACAGTGGCAGTTAGAGCATC	Diagnostic PCR for *PratfA* deletion
atf-RT-R	GTCATAGCTAACTCTACTCAAGACTCG	Diagnostic PCR for *PratfA* deletion
OEatf-5F-F	CATCATACCGATTACGCTCTAGG	Up flanks' amplification for *PratfA* overexpression
OEatf-5F-R	GCAGCTTGACTAACAGCTACCCCGCTTGAGCAGACATCACCATGAAGCCCCCGACACGA	Up flanks' amplification for *PratfA* overexpression
gpdA-R	GGTGATGTCTGCTCAAGCG	Promoter *gpdA* amplification
gpdA-F	TTTTTACAACTCTCCTATGAGTCGTTTACCCAGAATGCACCCGATAGCTCTGCAAAGGG	Promoter *gpdA* amplification
OEatf-3F-F	TCCTTCAATATCATCTTCTGTCGAGGCCTGATCATCGATGATGCGTCAAAATGTGGTGG	Down flanks' amplification for *PratfA* overexpression
OEatf-3F-R	CTGCACATAGATTGTGGCAAG	Down flanks' amplification for *PratfA* overexpression
gpdA-det-F	CGTCCAAATATCGTGCCTCTC	Diagnostic PCR for *PratfA* overexpression

Table S2. Recombinant strains in this study

Fungal Strain	Marker	Genetic Description
TYZHS12	*hph*	Δ*Pratf1*::*hph*
TYZHS20	*hph*	P_{gpdA}::*PratfA*::*hph*

Supplementary Figures

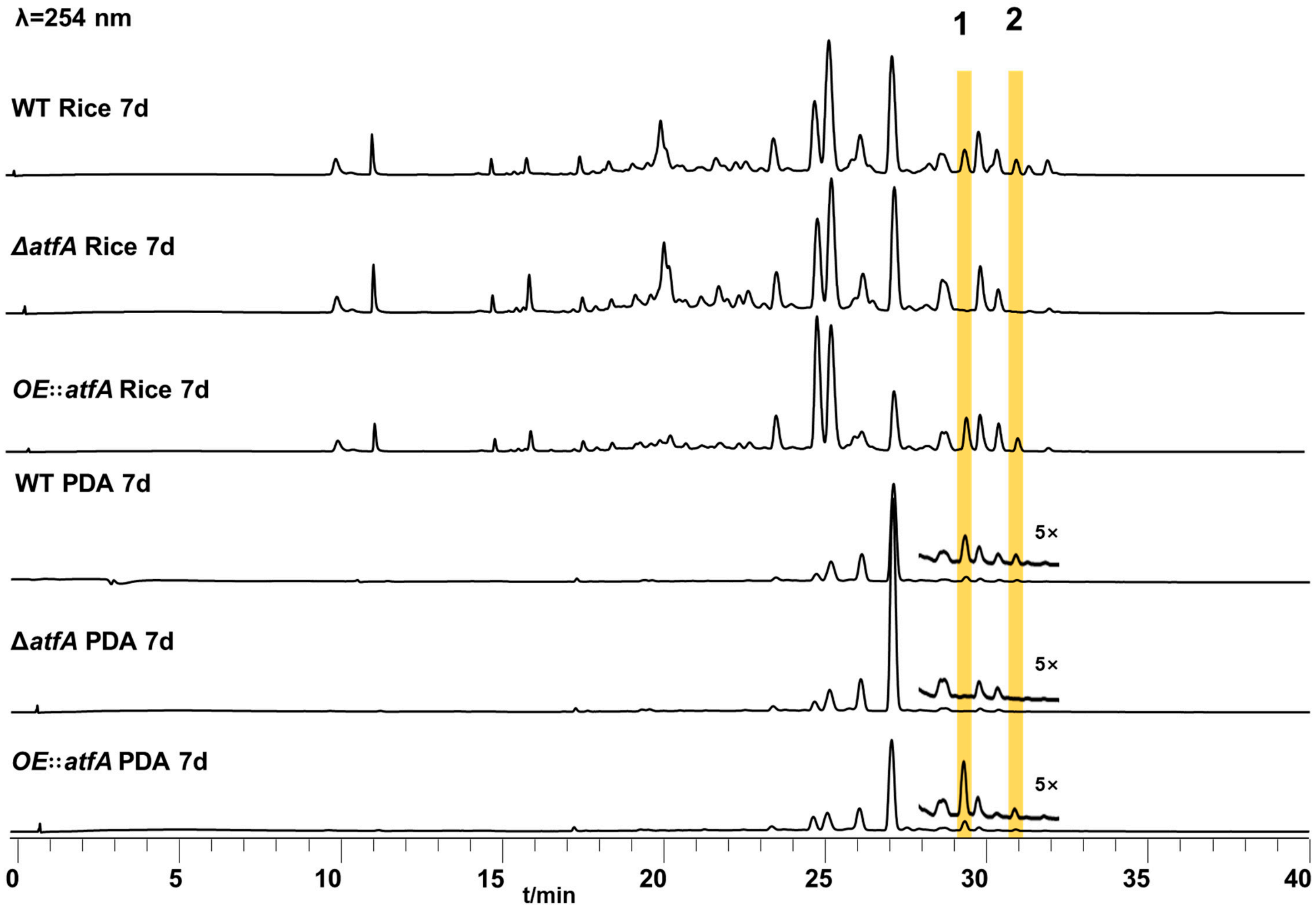

Figure S1 The HPLC analysis of WT, *ΔPratfA*, and the *OE::PratfA* mutants.

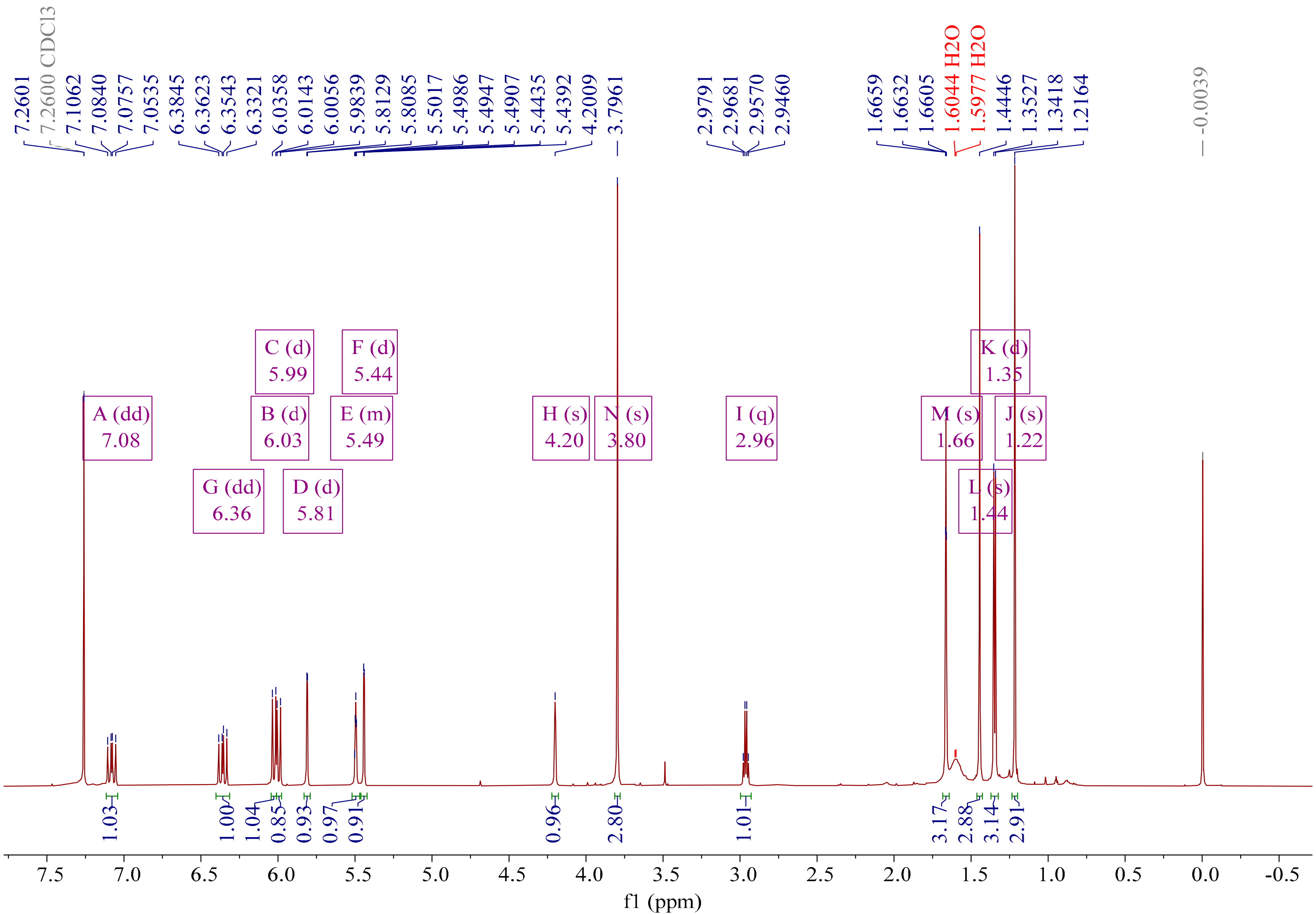

Figure S2 ^{1}H-NMR spectrum (500 MHz) of raistrilideA (**1**).

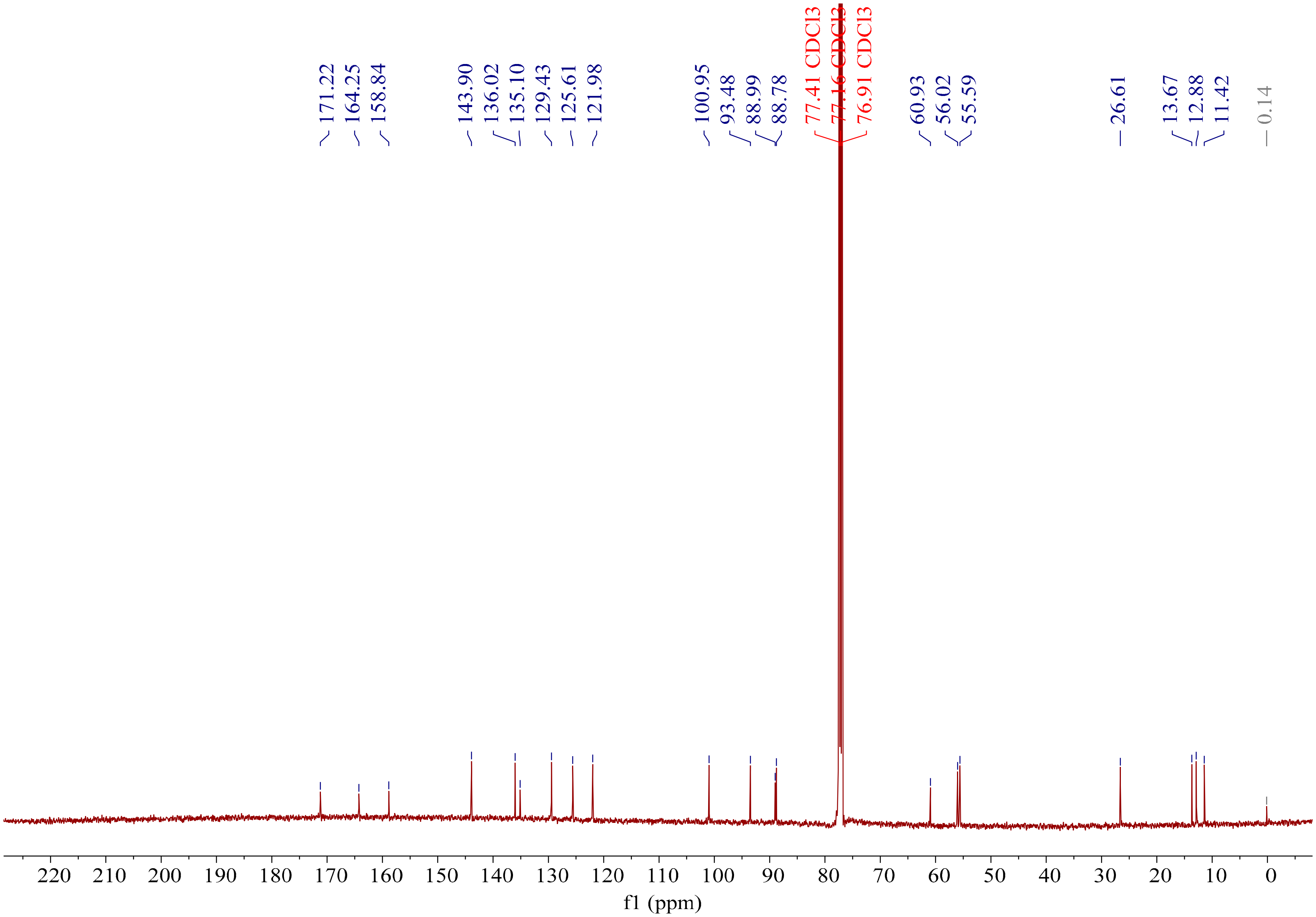

Figure S3. ^{13}C-NMR spectrum (125 MHz) of raistrilideA (**1**).

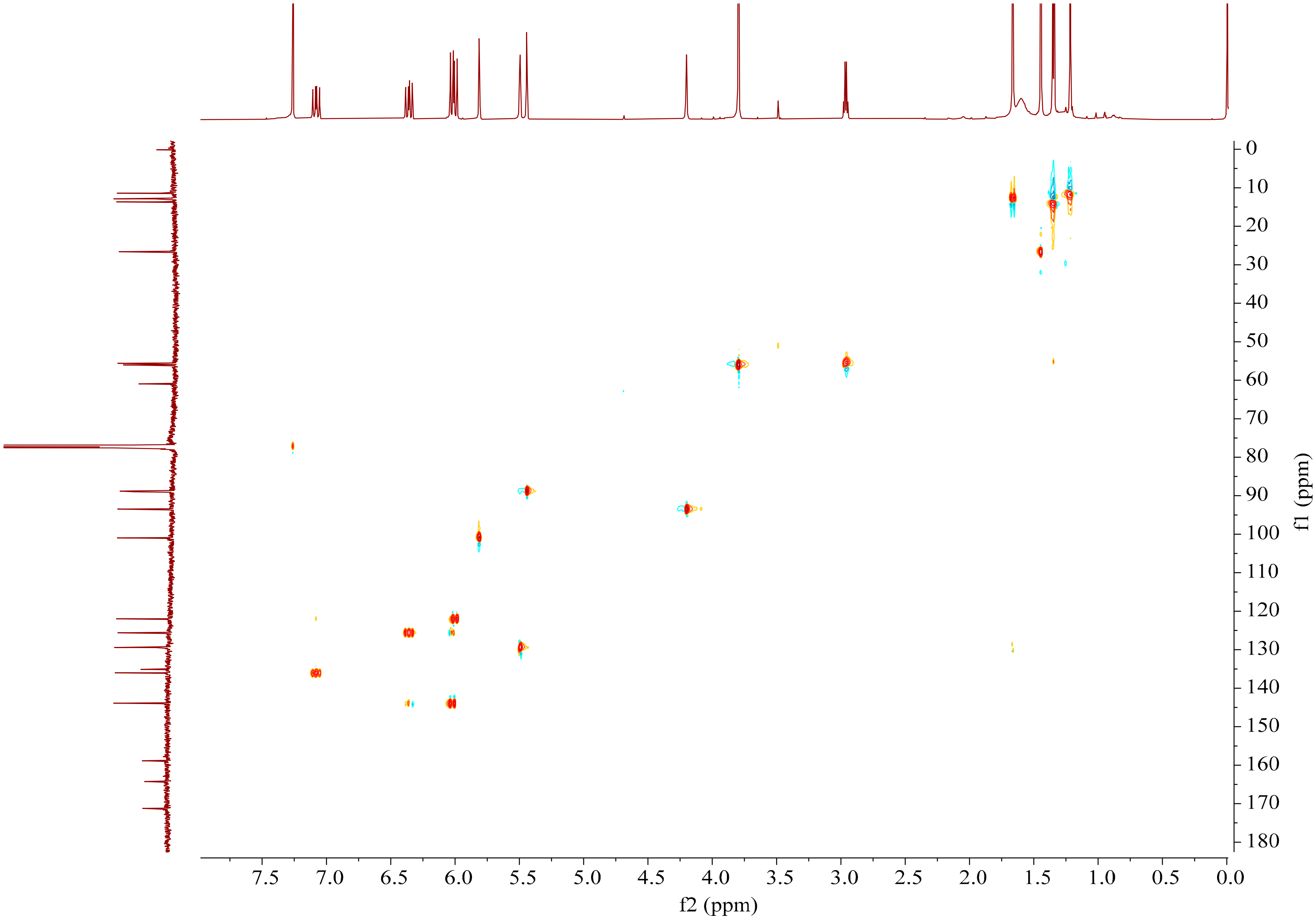

Figure S4. HSQC spectrum of raistrilideA (**1**).

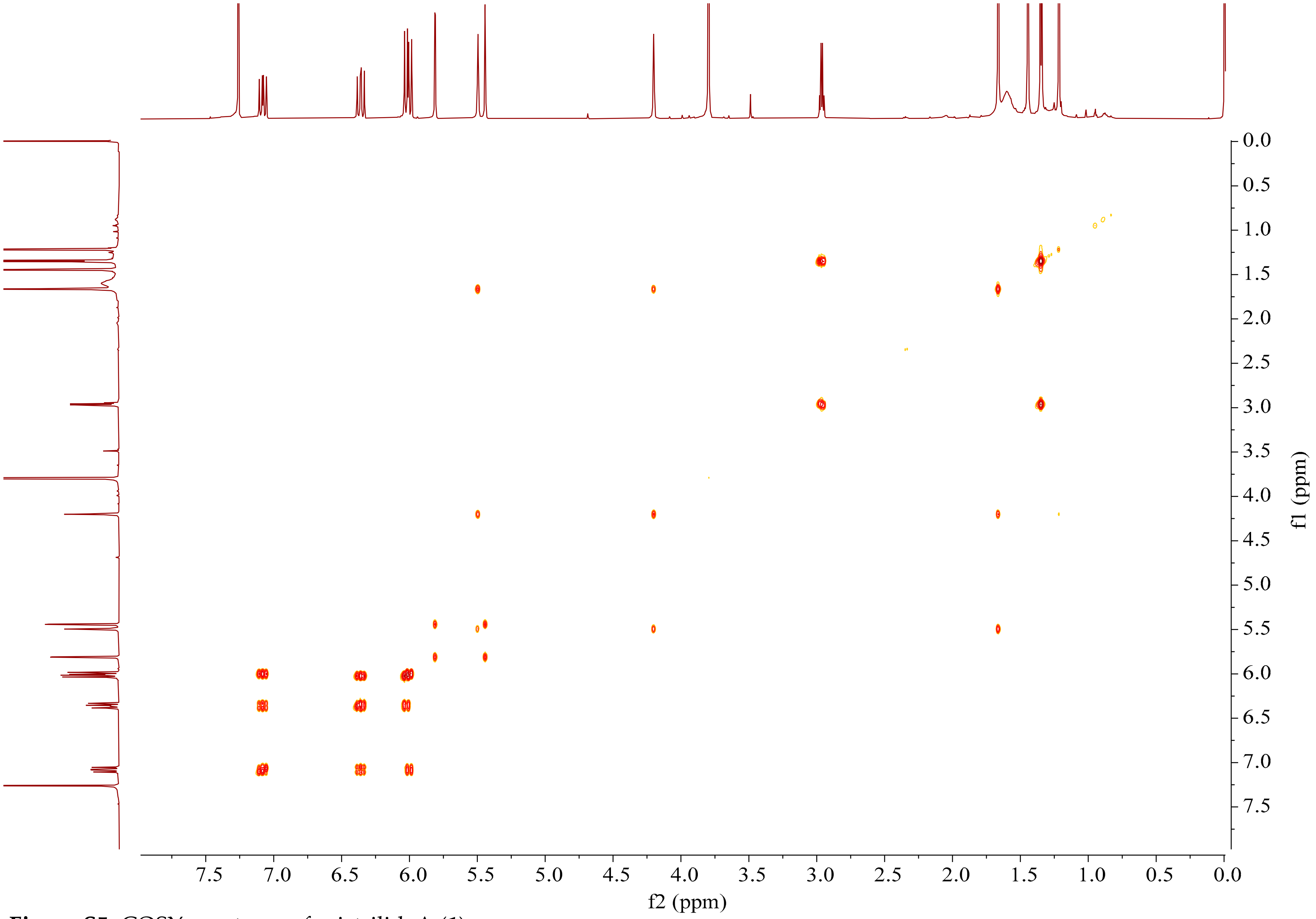

Figure S5. COSY spectrum of raistrilideA (**1**).

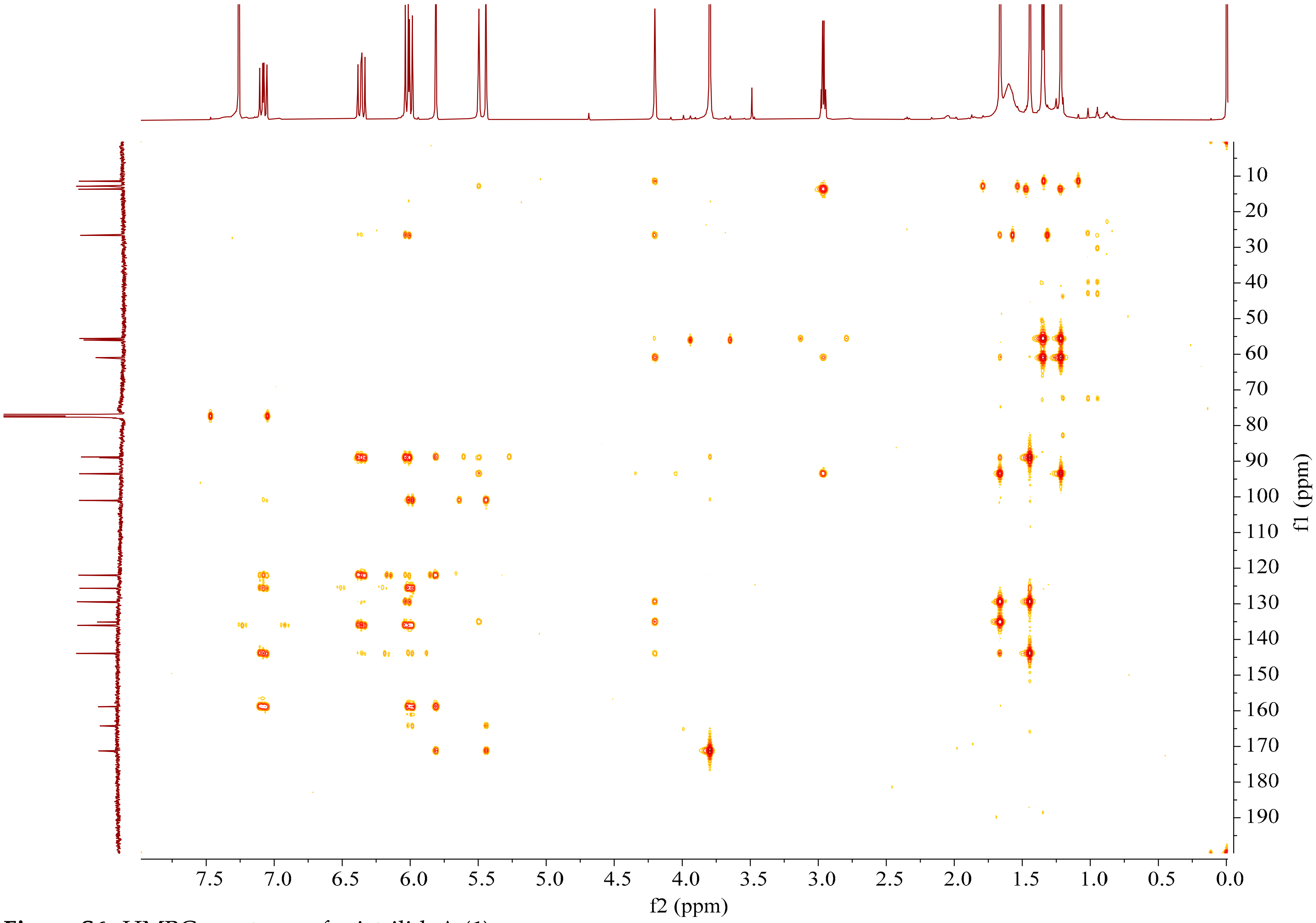

Figure S6. HMBC spectrum of raistrilideA (**1**).

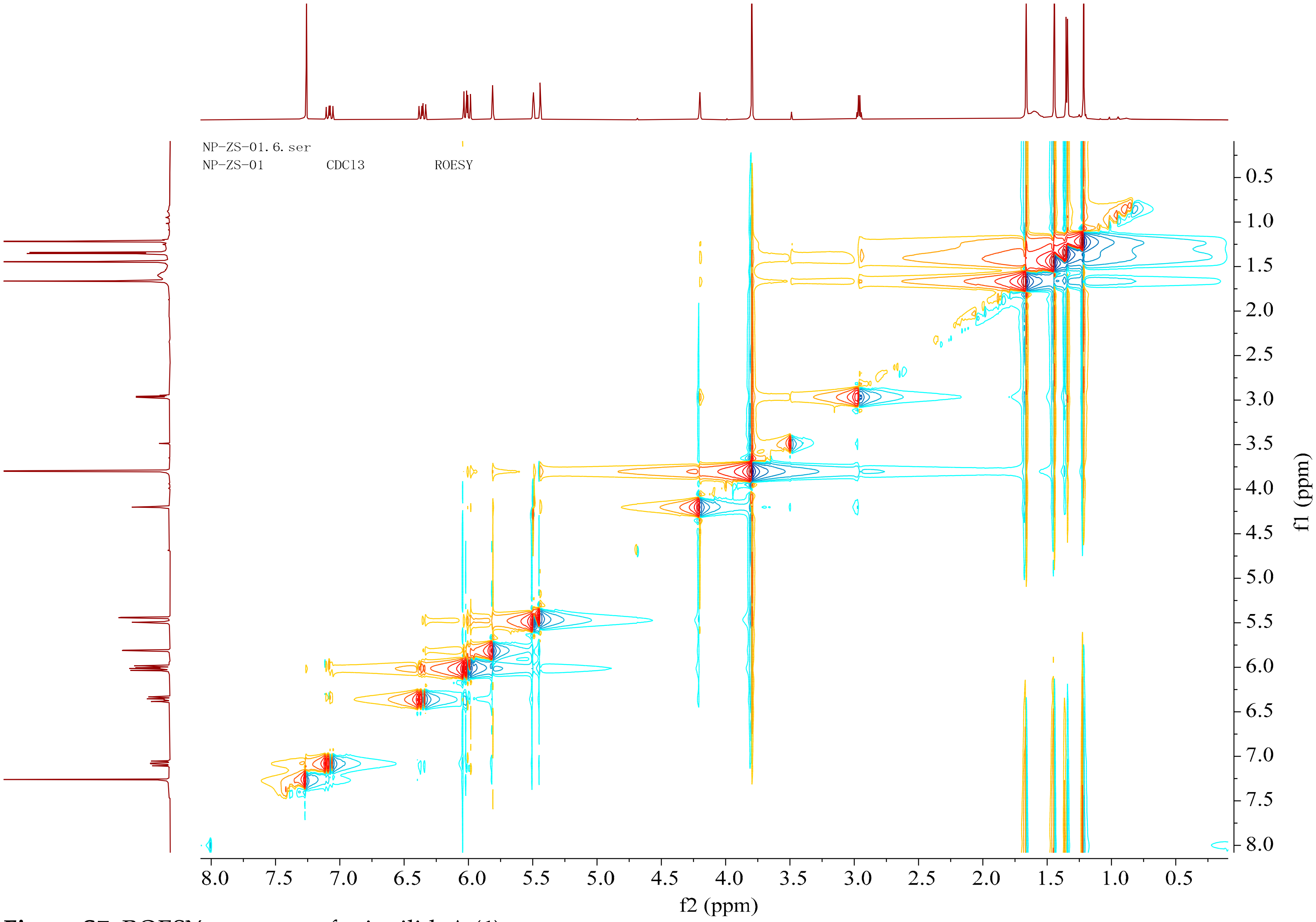

Figure S7. ROESY spectrum of raistrilideA (**1**).

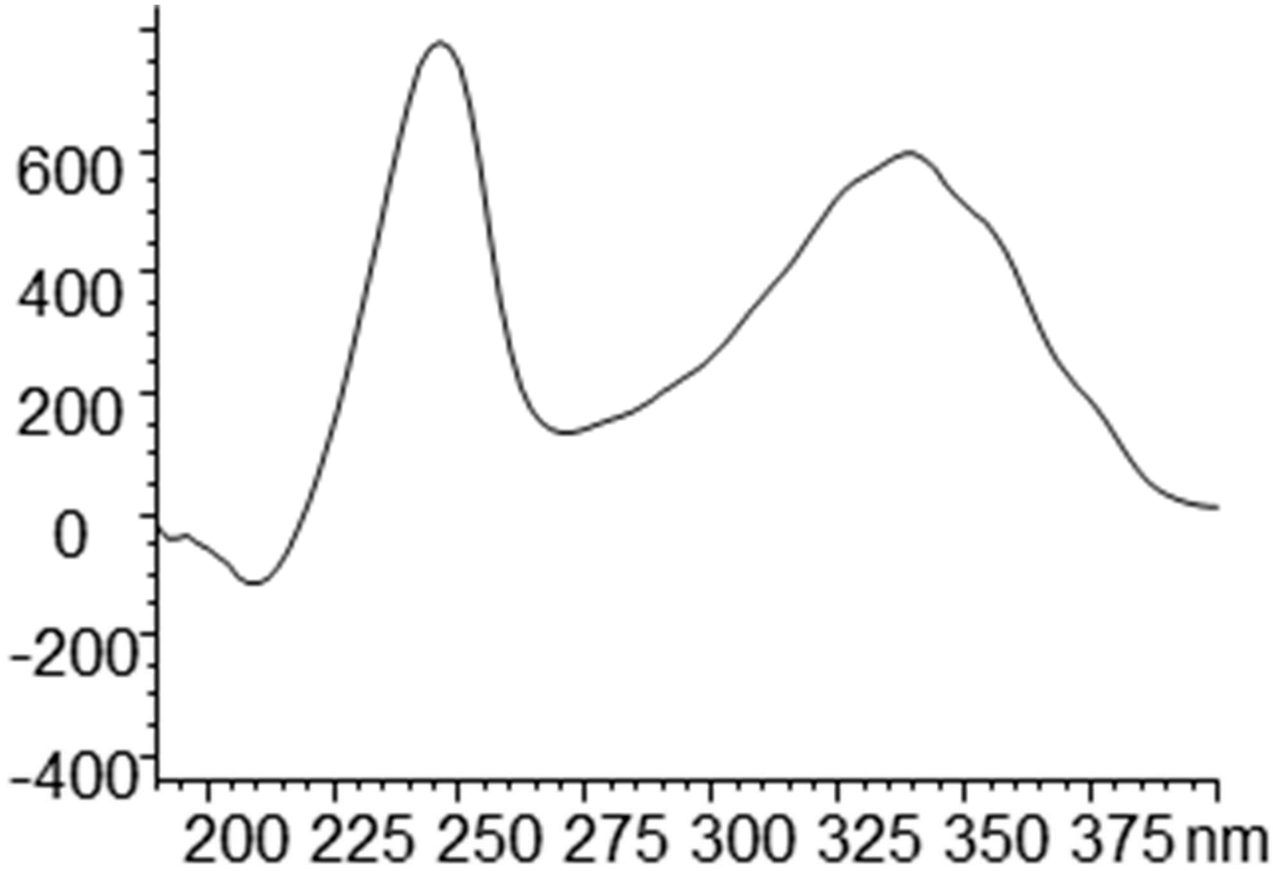

Figure S8. UV spectrum of raistrilideA (**1**).

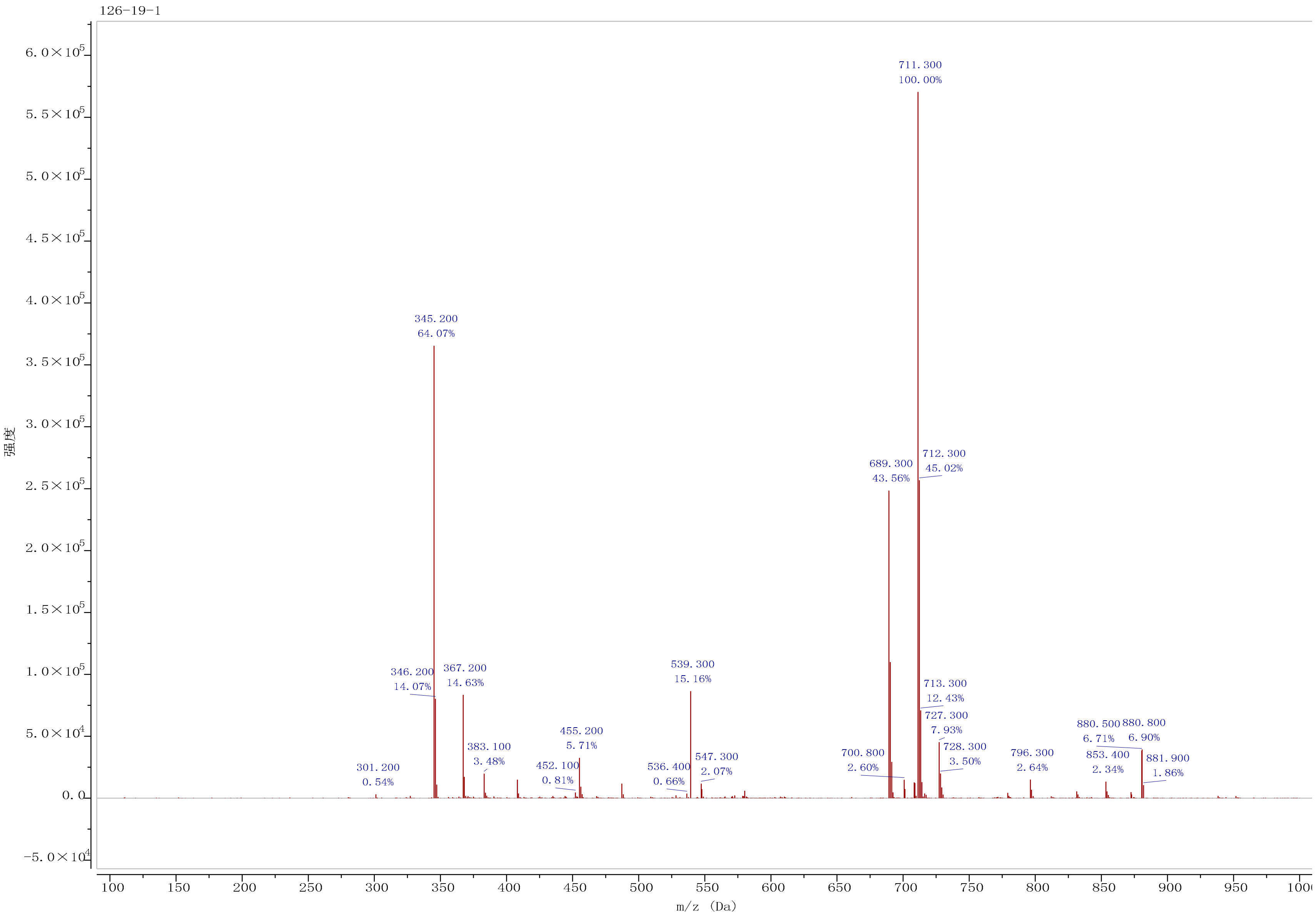

Figure S9. ESI mass spectrum of spectrum of raistrilideA (**1**)

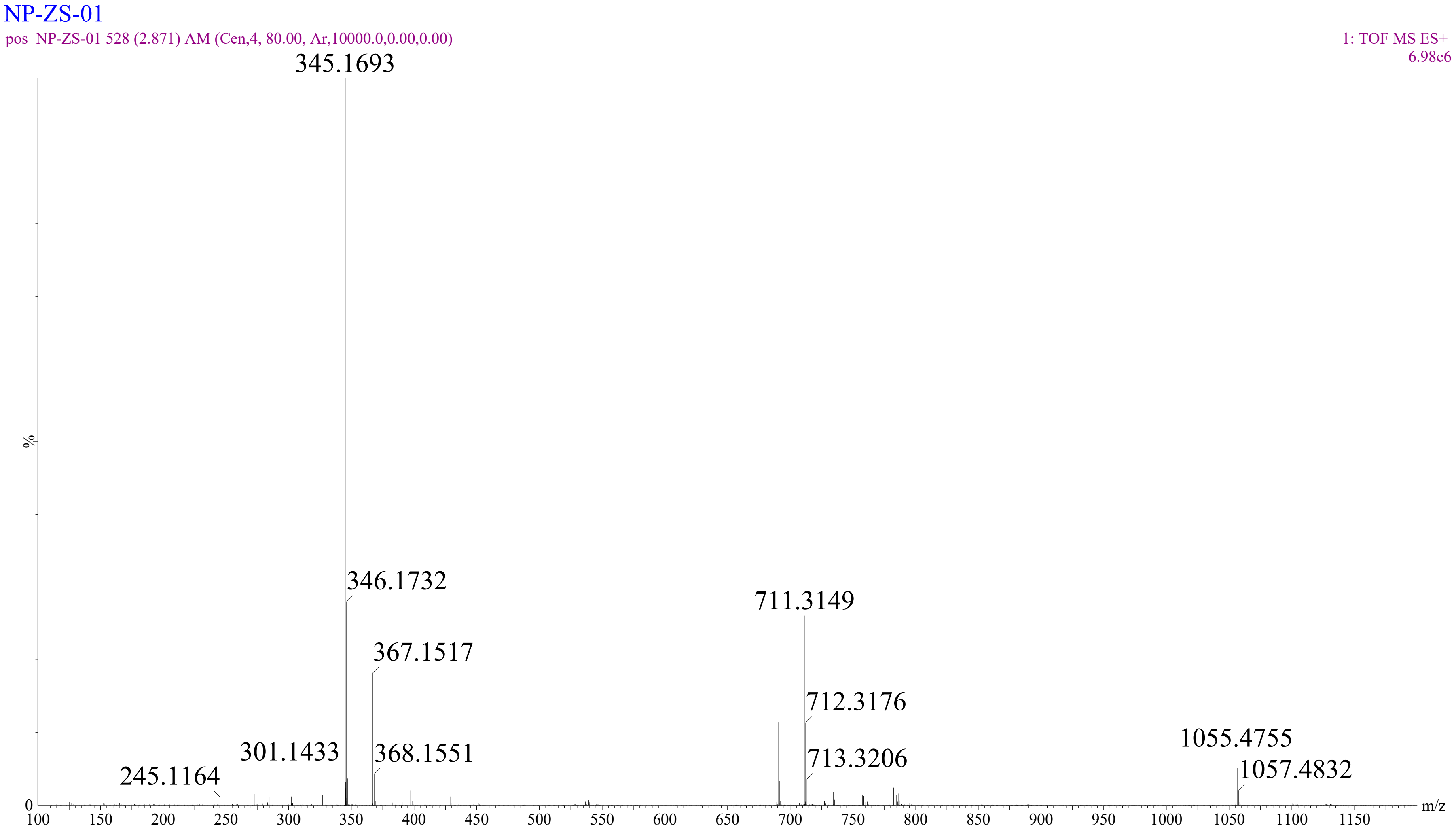

Figure S10. High resolution mass spectrum of spectrum of raistrilideA (**1**)
